# Accurate Noninvasive Assessment of Myocardial Iron Load in Advanced Heart Failure Patients

**DOI:** 10.1155/2020/8885189

**Published:** 2020-11-07

**Authors:** Przemysław Leszek, Barbara Sochanowicz, Kamil Brzóska, Leszek Kraj, Mariusz Kuśmierczyk, Witold Śmigielski, Tomasz M. Rywik, Małgorzata Sobieszczańska-Małek, Piotr Rozentryt, Marcin Kruszewski

**Affiliations:** ^1^The National Cardinal Stefan Wyszynski Institute of Cardiology, 04-628 Warszawa, Alpejska 42, Poland; ^2^Center of Radiobiology and Biological Dosimetry, Institute of Nuclear Chemistry and Technology, ul. Dorodna 16, 03-195 Warszawa, Poland; ^3^Department of Hematology, Oncology and Internal Diseases, The Medical University of Warsaw, 1A Banacha Str., 02-097 Warsaw, Poland; ^4^3rd Department of Cardiology, School of Medicine with the Division of Dentistry in Zabrze, Silesian Centre for Heart Disease, Medical University of Silesia in Katowice, 41-800 Zabrze Marii Skłodowskiej-Curie 9, Poland; ^5^Department of Toxicology and Health Protection, School of Public Health in Bytom, Medical University of Silesia, Katowice, 41-902 Bytom, Piekarska 18, Poland; ^6^Department of Medical Biology and Translational Research, Institute of Rural Health, ul. Jaczewskiego 2, 20-090 Lublin, Poland

## Abstract

**Background:**

Heart failure patients presenting with iron deficiency can benefit from systemic iron supplementation; however, there is the potential for iron overload to occur, which can seriously damage the heart. Therefore, myocardial iron (M-Iron) content should be precisely balanced, especially in already failing hearts. Unfortunately, the assessment of M-Iron via repeated heart biopsies or magnetic resonance imaging is unrealistic, and alternative serum markers must be found. This study is aimed at assessing M-Iron in patients with advanced heart failure (HF) and its association with a range of serum markers of iron metabolism.

**Methods:**

Left ventricle (LV) myocardial biopsies and serum samples were collected from 33 consecutive HF patients (25 males) with LV dysfunction (LV ejection fraction 22 (11) %; NT-proBNP 5464 (3308) pg/ml) during heart transplantation. Myocardial ferritin (M-FR) and soluble transferrin receptor (M-sTfR1) were assessed by ELISA, and M-Iron was determined by Instrumental Neutron Activation Analysis in LV biopsies. Nonfailing hearts (*n* = 11) were used as control/reference tissue. Concentrations of serum iron-related proteins (FR and sTfR1) were assessed.

**Results:**

LV M-Iron load was reduced in all HF patients and negatively associated with M-FR (*r* = −0.37, *p* = 0.05). Of the serum markers, sTfR1/logFR correlated with (*r* = −0.42; *p* = 0.04) and predicted (in a step-wise analysis, *R*^2^ = 0.18; *p* = 0.04) LV M-Iron. LV M-Iron load (*μ*g/g) can be calculated using the following formula: 210.24–22.869 × sTfR1/logFR.

**Conclusions:**

The sTfR1/logFR ratio can be used to predict LV M-Iron levels. Therefore, serum FR and sTfR1 levels could be used to indirectly assess LV M-Iron, thereby increasing the safety of iron repletion therapy in HF patients.

## 1. Introduction

Iron plays a crucial role in oxygen transport and storage, cardiac and skeletal muscle metabolism, energy production, and protein synthesis [1]. Iron deficiency (ID) is a common comorbidity in cardiac patients, particularly in heart failure (HF) patients, resulting in further detrimental effects [2]. Recent clinical studies have demonstrated that in HF patients presenting with ID, iron supplementation can lead to significant clinical improvement [3–5]. As such, the 2016 European Society of Cardiology (ESC) HF guidelines state that iron replacement therapy should be considered in HF patients with ID [6]. This clinical benefit of intravenous (iv) iron supplementation in HF patients seems to be independent of the presence of anemia [1, 7]. Despite these benefits, an excess of iron could potentially exert harmful effects; for example, improperly shielded iron ions can catalyze the production of reactive free radicals (due to the rapid oxidation-reduction cycling between Fe^3+^ and Fe^2+^ states), resulting in oxidative damage [8–12]. Therefore, patient iron levels should be closely monitored during iron replacement therapy.

Myocardial iron (M-Iron) metabolism has also been shown to be strongly related to left ventricle (LV) remodeling in HF [13, 14]. Indeed, based on an experimental rat model, we know that ID-anemia leads to molecular heart remodeling and, finally, LV dilatation [1]. These findings align with our data on human explanted hearts, which show a significant reduction in M-Iron load in failing LVs [13–15]. Hence, iron replenishment may be beneficial for the heart [16–18]. Conversely, low iron concentrations have been shown to exert positive effects by stimulating inducible nitric oxidase synthase (iNOS) activity and nitric oxide (NO) production, which promote cell survival in cardiomyocytes [17]. As iron replenishment can exert both beneficial and detrimental effects on a failing myocardium, depending on the actual M-Iron content, proper characterization of the M-Iron load is a key, especially in HF subjects. However, to date, there are no standardized criteria for monitoring the effectiveness and safety of iv iron treatment on the myocardium.

This study is aimed at assessing M-Iron load in the failing LV in relation to serum markers of iron metabolism to develop an indirect method of M-Iron assessment without performing a heart biopsy. This noninvasive assessment should increase the safety of iron supplementation in HF patients.

## 2. Material and Methods

### 2.1. Study Population and Protocol

The protocol was approved by the Local Ethics Committee. Each patient participating in the study signed an informed consent form after a detailed explanation of the study principles. The study group comprised 33 consecutive patients referred to orthotopic heart transplantation (OHT). Myocardial studies were performed in failing ventricular myocardium obtained during transplantation.

### 2.2. Study Protocol

All clinical assessments and blood sampling were performed just before OHT.

Two-dimensional, M-mode, and color Doppler transthoracic echocardiography was performed at rest according to the recommendations of the American Society of Echocardiography. Right heart catheterization, hemodynamic and cardiac output measurements, and resistance calculations were performed just before OHT.

Blood counts were determined with an automatic counter (Sysmex K4500), as follows: red blood cell (RBC) count (normal range, male/female: 4.6–6.2/4.2–5.4 million/*μ*l), hematocrit (Hct: 42–52/37–47%), mean corpuscular volume (MCV: d80–99 fl), hemoglobin (Hb: 14–18/12–16 g/dl), and mean corpuscular hemoglobin (MCH: 27–32 pg).

Body iron status and biochemical assessment were evaluated in serum using the Clinical Chemistry System Olympus 680 (Olympus Life Science) as follows: serum iron (normal range, male/female: 70–180/60–180 *μ*g/dl), transferrin (200–360 mg/dl), transferrin saturation (TSAT, calculated from serum iron/transferrin: 15–45%), total iron-binding capacity (TIBC, calculated from iron: 210–340/260–390 *μ*g/dl), and unsaturated iron-binding capacity (UIBC, calculated from TIBC and iron: 140–180/200–210 *μ*g/dl). The COBAS Integra® 800 System (Roche Diagnostic) was used to evaluate soluble transferrin receptor (sTfR: 2.2–5.0/1.9–4.4 mg/l), high-sensitivity C-reactive protein (hsCRP, normal range: 0–0.5 mg/dl), sodium (136–145 mmol/l), and creatinine (62–106/44–80 *μ*mol/l). The ARCHITECT® Immunochemistry Diagnostics Platform (Abbott Laboratories) was used to determine ferritin (FR, normal range, male/female: 4.63–204/21.81–274.66 ng/ml). Chemiluminescent IMMULITE 2000 (Siemens Healthcare Diagnostics) was used to measure erythropoietin (EPO, normal range, 3.7–29.5 mIU/ml). The Cobas e411R analyzer (Roche Diagnostic) was used to determine N-terminal pro-B-type natriuretic peptide (NT-proBNP, normal range, 0–125 pg/ml). Tumor Necrosis Factor-alpha (TNF*α*) levels were assayed by an enzyme-linked immunosorbent assay (ELISA, normal ranges < 8 pg/ml) according to the manufacturer's instructions (Human TNF-alpha, R&D System Inc., USA).

The estimated total dose required for iron repletion (TIRD) was assessed by the Ganzoni formula: TIRD (mg) = body weight (kg) × (target Hb–actual Hb in g/l) × 2.4^∗^ + iron depot (mg)^∗∗^ (^∗^the factor 2.4 = 0.0034 × 0.07 × 10 000;  ^∗∗^iron depot:<35 kg body weight : iron depot = 15 mg/kg body weight; ≥35 kg body weight : iron depot = 500 mg).

### 2.3. Myocardial Assessments

Tissue samples of the LV free wall were taken at the time of explantation (avoiding scarred, fibrotic, or adipose tissue, endocardium, epicardium, or great vessels), rinsed immediately, blotted dry, frozen in liquid nitrogen, and kept at -80°C until use.

#### 2.3.1. Myocardial Ferritin (M-FR) and Myocardial Soluble Transferrin Receptor (M-sTfR1) Assessment

In total, 80–100 mg of cardiac tissue was homogenized using an Ultra-Turrax T25 homogenizer in buffer with a Complete Protease Inhibitor Cocktail. Homogenate was filtered through two layers of gauze and centrifuged at 10,000 × g for 10 min. The supernatant was collected, portioned, rapid frozen in liquid nitrogen, and stored at -75°C. The total protein concentration was determined by the Bradford method. M-FR (kit from Alpha Diagnostic International Inc., San Antonio, TX, USA) and M-sTfR1 (kit from BioVendor GmbH, Heidelberg, Germany) were assayed by ELISA according to the manufacturer's instructions.

#### 2.3.2. Myocardial Total Iron (M-Iron) Assessment

M-Iron was assayed by Instrumental Neutron Activation Analysis (INAA). In brief, frozen samples were lyophilized (Freezemobile 12XL, Virtis Company, New York, US), weighed, and packaged in HDPE snap-cap capsules (Faculteit Biologie, Vrije Universiteit, Amsterdam, Holland). The certified reference material NIST 1577c Bovine Liver (National Institute of Standards and Technology (NIST), US) was used for quality control. Samples and standards were irradiated at the neutron flux of 10^14^ cm^−2^ s^−1^ for 50 min in a nuclear reactor MARIA (Świerk, Poland). After three weeks of cooling, the gamma-ray emission of the samples and standards was measured with the GENIE-2000 Canberra Gamma Spectrometry System and the GENIE 2000 software (Canberra Industries, Inc., Meriden, US).

### 2.4. Statistical Analysis

Data are expressed as means (SD) or as medians (IQR) for data that were not normally distributed. The test for normality for each analyzed parameter was performed using the Shapiro-Wilk test. Pearson correlation matrices were used to establish univariate correlations among M-Iron and other parameters. A stepwise multiple regression analysis was employed to assess the strongest model of independent predictors of M-Iron.

## 3. Results

### 3.1. Baseline Characteristics of the Study Group

The study group consisted of 33 consecutive, symptomatic HF patients (25 males), with a mean age of 48 years, who were referred for OHT. The study group presented with LV dilatation or dysfunction (LVESV 189 (95) ml; LVEDV 245 (83) ml; LVEF 22 (11) %), RV enlargement (RVD 32 (10) mm), pulmonary hypertension (PVR 3.36 (1.2) W.u.), and significant neurohumoral (NT-proBNP 5464 (3308) pg/ml) and proinflammatory (TNF*α* 15.8 (9.7) pg/ml; hsCRP 0.72 (0.3) mg/dl) activation (Table 1).

### 3.2. Iron- and HF-Related Parameters Associated with M-Iron in the Failing LV

M-Iron load at the cellular level was recently shown to be reduced in the failing heart, without significant changes in the expression of M-FR and M-sTfR1 [13, 14]. However, in patients with M-Iron deficiency, M-Iron reduction is accompanied by decreased M-FR expression [13, 14].

In our current calculations, based on Pearson's correlation matrices, we found that in the failing LV myocardium, M-Iron load was negatively associated with M-sTfR1 but not M-FR (Table 2).

Although serum sTfR1 tended to correlate negatively with M-Iron, only the sTfR1/logFR ratio was significantly negatively associated with M-Iron. However, we did not confirm any correlation between M-Iron and other parameters routinely utilized for iron metabolism assessment (Table 3). Furthermore, we did not prove any association among M-Iron and TIRD calculated according to the Ganzoni formula or other RBC-related parameters, except RBC number (Table 3). Finally, neither the degree of LV dysfunction nor the level of neurohumoral or proinflammatory activation (i.e., two parameters frequently used to assess the severity of HF) was associated with M-Iron (Table 3).

### 3.3. Predictive Value of the sTfR1/logFR Ratio in Assessing M-Iron Load

**Figure 1:**
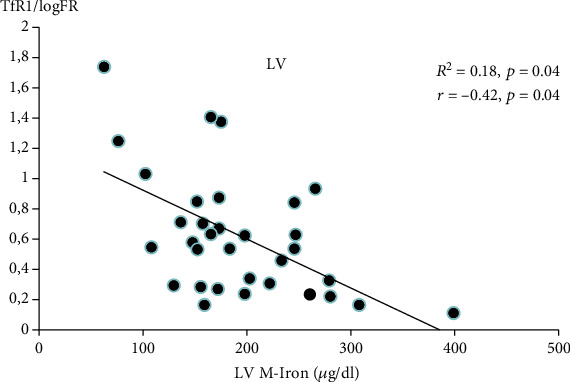
Independent predictors of myocardial iron load (M-Iron) (in failing left ventricle). LV M-Iron: left ventricle myocardial iron load; sTfR1/log FR: serum soluble transferrin receptor 1/log ferritin.

Among all iron- and RBC-related parameters described above, only the sTfR1/logFR ratio was an independent predictor of M-Iron (Figure 1).

Based on our calculations and obtained correlations after the mathematical transformation, the formula for LV M-Iron calculations is LV M‐Iron load (*μ*g/g) = 210.24–22.869 × sTfR1/logFR.

We previously compared M-Iron content in LV myocardium from HF and non-HF subjects [14] and found that the normal LV iron content ranges from 200 to 300 *μ*g/g. Therefore, using the above equation, the normal M-Iron ranges from 200 *μ*g/g, which corresponds to a sTfR1/logFR of 0.753, and 300 *μ*g/g, which corresponds to a sTfR1/logFR of 0.01.

## 4. Discussion

ID (with or without anemia) is common in chronic diseases, and iv iron supplementation is now often used in cardiology, oncology, hematology, and nephrology patients [1, 3–7, 19, 20]. However, an excess of iron in the body leads to dysfunctions of many organs, including the heart. Thalassemia, sickle cell anemia, and hemochromatosis are the most frequently occurring diseases with altered iron homeostasis, leading to uncontrolled iron entry and progressive tissue damage due to intracellular oxidative stress arising from the excessive production of free radicals [8–10, 12]. Therefore, when supplementing iron (especially iv), monitoring of iron stores is necessary to avoid overcorrection, which may lead to M-Iron overload.

Bone marrow biopsy assessment is the most accurate method to define ID (i.e., the depletion of iron in bone morrow stores) [21, 22]. However, performing a bone marrow biopsy simply to define ID is not appropriate. Similarly, a heart biopsy cannot be used routinely to evaluate the M-Iron load. Therefore, in real life, ID is defined based on laboratory assessments, including serum FR levels of *<*100 *μ*g/l or between 100 and 299 *μ*g/l when TSAT is *<*20% [23, 24]. Nevertheless, although this definition is widely accepted and the examinations are easy to perform, they do not accurately reflect the iron load in the body [25].

Regarding M-Iron, there is a general agreement that the M-Iron load is reduced in a failing LV [13–15]. Moreover, in HF patients with ID, the expression of the main iron storage protein, M-FR, is also reduced [13, 14]. Although we found no significant correlation between M-Iron and M-FR in our study, it is important to note that M-FR expression is not exclusively related to M-Iron load but also to inflammation and oxidative stress that accompanies the HF syndrome [24].

Only a few studies have evaluated changes in the expression of M-TfR1, the main protein responsible for iron acquisition, albeit with conflicting results. While Maeder et al. reported a reduction of M-TfR1 expression at the mRNA level in HF [15], we were unable to prove this finding at the protein level [13, 14]. However, we found a significant negative correlation between M-Iron load in the failing LV myocardium and M-TfR1 protein expression that is in agreement with the known role of TfR1 in iron metabolism.

Although there are undoubtedly links between M-Iron, M-FR, and M-TfR1 at the cellular level in the failing myocardium, our work proves that traditionally used clinical serum markers for body iron stores, such as TSAT and FR, do not reflect the actual M-Iron status. Serum FR has been commonly used as a clinical biomarker of ID since the early 1970s [26]. FR is produced in response to an increase in cellular iron content and reflects the storage compartment of cellular iron. However, increased levels of FR are observed at the onset of acute and chronic diseases [24, 26]. Thus, the diagnostic utility of FR in the HF population may be compromised by a false-positive increase in FR in these conditions. In turn, transferrin is a negative acute-phase reactant, and reduced TSAT levels are also observed in chronic conditions [27]. Moreover, TSAT levels show circadian fluctuations and are related to sleep quality [28]. Indeed, Nanas et al. [21] previously defined ID based on bone morrow assessment and proved that serum FR was not a reliable marker of ID in HF patients. In the case of iron deficiency for hematopoiesis, RBC correlates with the classic biochemical parameters of iron metabolism (TSAT, TIBC, and FR) in the blood serum. In this study, LV M-Iron does not correlate with these biochemical parameters. Interestingly, we observed a negative correlation between LV M-Iron and RBC, which proves the compartmentalization of iron in different tissues. However, the exact mechanisms that regulate the interaction between these compartments are unclear.

As the currently used serum markers are not satisfactory, and the direct assessment of M-Iron content by heart biopsies or frequent magnetic resonance imaging examination is not realistic, alternative serum markers must be found for the reliable assessment of M-Iron content. Our results validated the parameters commonly used for iron load assessment and demonstrated that the M-iron load can be assessed more accurately based on serum sTfR1, particularly by calculating the sTfR1/logFR ratio. Circulating sTfR1 is produced by the proteolytic cleavage in direct proportion to the cellular receptor content [29]. sTfR1 levels reflect the total body mass of receptors, whereas the rate of their synthesis is closely linked to the iron requirements of the cells. In contrast to FR and TSAT, acute-phase reactions do not influence the sTfR1 serum level [30]. The most common cause of elevated serum sTfR1 levels is erythropoiesis in the bone marrow [29]; however, it can be released from other tissues, including cardiac [31]. As sTfR1 and FR levels reflect the functional and storage iron compartments, respectively, the sTfR1/logFR ratio has been suggested as a parameter for estimating iron status in the human body [32, 33]. Indeed, Enko et al. showed that the sTfR1/logFR ratio is superior to sTfR1, FR, and TSAT in predicting functional ID in hospitalized patients, irrespective of the acute-phase reaction [25, 34]. Our results also show the accuracy of sTfR1/logFR in the proper characterization of M-Iron load and homeostasis.

In the HF population, iron repletion and the total iron repletion dose (TIRD) required for supplementation for an individual ID patient is usually calculated using the Ganzoni formula and relies on the subject's weight and Hb value [35]. However, besides the RBC count, we (and others [36]) found no association between M-iron and TIRD or the other RBC-related parameters in the Ganzoni formula.

Iron repletion therapy is usually tailored based on the presence of ID criteria (i.e., serum FR and TSAT levels) [4, 37]. We show that among all frequently used iron/RBC parameters, only sTfR1/logFR is an independent predictor of M-Iron. We determined that the best formula for LV M-Iron calculations is LV M‐Iron load (*μ*g/g) = 210.24–22.869 × sTfR1/logFR. Therefore, when referring to the LV M-Iron load in HF and non-HF patients, we postulate that iv iron repletion therapy may be additionally tailored by the noninvasive M-Iron calculation based on the above formula. This formula allows the LV M-Iron content to be approximated at each stage of iv iron supplementation in a noninvasive way.

There are some limitations to our study. In particular, the presented work is based on a limited, but representative, homogeneous population of patients with advanced HF (33 patients) subjected to heart transplantation. Despite this, the population size is comparable to the size of the population on which the fundamental formula for iron deficiency calculations was established by Ganzoni (30 patients). Nonetheless, prospective longitudinal studies involving follow-up measurements of proposed parameters are needed for improved data modeling.

In summary, among the commonly used serum markers for iron turnover assessment, only the sTfR1/logFR ratio is an independent predictor of LV M-Iron. In this study, we present a formula that enables the indirect assessment of LV M-Iron, which will help increase the safety of iron repletion therapy in HF patients.

## Figures and Tables

**Table 1:** Clinical characteristics of the study group.

	Heart failure patients (*n* = 33)
Age (yrs)	51 (6.5)
Men/women, *n*	25/8
Etiology: idiopathic/ischemic/other, *n*	10/21/2
NYHA functional class: III/IV, *n*	15/18
LVESV (ml)/LVEDV (ml)/LVEF (%)	189 (95)/245 (83)/22 (11)
RVD (mm)	32 (10)
Mean PWP/mean PAP (mmHg)	23 (9)/33 ± 13
PVR/SVR (W.u.)	3.36 (1.2)/21.9 (6.2)
CI (l/min/m^2^)	1.93 (0.64)
Red blood cells (ml/*μ*l)/hematocrit (%)	4.5 (0.6)/39.7 (5.3)
Mean corpuscular volume (fl)	88.4 (7.1)
Hemoglobin (g/dl)/mean corpuscular hemoglobin (pg)	13.2 (1.7)/29.5 (2.5)
Serum iron (*μ*g/dl)	62 (32)
Serum transferrin (mg/dl)/transferrin saturation (%)	240 (47)/19.5 (10.6)
Total iron-binding capacity/unsaturated iron-binding capacity (*μ*mol/l)	288 (56)/41 (11)
Serum soluble transferrin receptor (mg/l)	3.2 (2.6)
Serum ferritin (ng/ml)	156 (122)
Erythropoietin (mIU/ml)	29.5 (44.4)
TIRD (see Material and Methods) (mg)	808 (323)
NT-proBNP (pg/ml)	5464 (3308)
TNF*α* (pg/ml)	15.8 (9.7)
hsCRP (mg/dl)	0.72 (0.3)
Serum sodium (mEq/l)	138 (2.5)
Serum creatinine (*μ*mol/l)	108 (35)

LVESV/LVEDV: left ventricle volume end-diastolic/systolic; LVEF: left ventricle ejection fraction; RVD: right ventricle diastolic size; PWP: mean pulmonary wedge pressure; PAP: mean pulmonary artery pressure; PVR: pulmonary vascular resistance; SVR: systemic vascular resistance; CI: cardiac index; TIRD: total iron dose required for iron repletion calculated using the Ganzoni formula (see Material and Methods); NT-proBNP: N-terminal pro-B-type natriuretic peptide; TNF*α*: Tumor Necrosis Factor-alpha; hsCRP: high-sensitivity C-reactive protein.

**Table 2:** Association between myocardial iron load and iron handling proteins in the failing left ventricle.

	M-FR	M-sTfR1
LV M-Iron	*r* = 0.01	*r* = −0.37
	*p* = 0.94	*p* = 0.05

LV M-Iron: left ventricle myocardial iron load; M-FR: myocardial ferritin; M-sTfR1: myocardial soluble transferrin receptor.

**Table:** (a) Associations with iron-related serum parameters

LV M-Iron	Iron	Transferrin	TSAT	TIBC	UIBC	sTfR1	FR	sTfR1/logFR
	*r* = −0.07	*r* = −0.17	*r* = −0.03	*r* = −0.19	*r* = −0.15	*r* = −0.38	*r* = 0.13	*r* = −0.42
	*p* = 0.77	*p* = 0.54	*p* = 0.90	*p* = 0.38	*p* = 0.49	*p* = 0.07	*p* = 0.54	*p* = 0.04

**Table:** (b) Associations with red blood cell-related parameters

LV M-Iron	TIRD	RBC	Hb	Hct	MCV	MCH	MCHC	Weight	EPO
	*r* = 0.16	*r* = −0.39	*r* = −0.22	*r* = −0.22	*r* = 0.23	*r* = 0.32	*r* = 0.11	*r* = −0.20	*r* = −0.29
	*p* = 0.42	*p* = 0.04	*p* = 0.25	*p* = 0.26	*p* = 0.25	*p* = 0.10	*p* = 0.56	*p* = 0.31	*p* = 0.18

**Table:** (c) Associations with HF severity-related parameters

LV M-Iron	LVESV	LVEDV	LVEF	RVD	mPWP	PVR	NT-proBNP	hsCRP	TNF*α*	Serum sodium	Creatinine clearance
	*r* = −0.26	*r* = −0.19	*r* = −0.18	*r* = −0.32	*r* = 0.23	*r* = 0.19	*r* = −0.22	*r* = −0.02	*r* = 0.09	*r* = −0.12	*r* = −0.03
	*p* = 0.20	*p* = 0.32	*p* = 0.36	*p* = 0.21	*p* = 0.54	*p* = 0.17	*p* = 0.34	*p* = 0.93	*p* = 0.69	*p* = 0.38	*p* = 0.85

LV M-Iron: left ventricle myocardial iron load. Iron-related serum parameters: iron: serum iron; TSAT: transferrin saturation; TIBC: total iron-binding capacity; UIBC: unsaturated iron-binding capacity; sTfR1: soluble transferrin receptor 1; FR: ferritin. Red blood cell-related parameters: TIRD: total iron dose required for iron repletion calculated using the Ganzoni formula; RBC: red blood cells; Hb: hemoglobin; Hct: hematocrit; MCV: mean corpuscular volume; MCH: mean corpuscular hemoglobin; MCHC: mean corpuscular hemoglobin concentration; EPO: erythropoietin. Heart failure severity-related parameters: LVESV/LVEDV: left ventricle volume end-diastolic/systolic; LVEF: left ventricle ejection fraction; RVD: right ventricle diastolic size; mPWP: mean pulmonary wedge pressure; PVR: pulmonary vascular resistance; NT-proBNP: N-terminal pro-B-type natriuretic peptide; hsCRP: high-sensitivity C-reactive protein; TNF*α*: Tumor Necrosis Factor-alpha.

## Data Availability

The data are not freely available, due to restricted access to human data related to legal (GDPR) and ethical concerns.
